# P-wave indices and left atrial mechanics as predictors of atrial cardiopathy in embolic stroke of undetermined source

**DOI:** 10.1038/s41598-023-44285-2

**Published:** 2023-11-15

**Authors:** Samia Masood, Syed Mohammad Kamil Ashraf, Mohammad Azharuddin Malik, Shagufta Wahab

**Affiliations:** 1 Department of Medicine, Jawaharlal Nehru Medical College and Hospital, Aligarh Muslim University, Aligarh, UP 202002 India; 2 Department of Cardiology, Jawaharlal Nehru Medical College and Hospital, Aligarh Muslim University, Aligarh, UP 202002 India; 3 Department of Radiodiagnosis, Jawaharlal Nehru Medical College and Hospital, Aligarh Muslim University, Aligarh, UP 202002 India

**Keywords:** Cardiology, Arrhythmias

## Abstract

Recent research has shed light on the culpability of LA (left atrial) abnormality, in the form of atrial cardiopathy, as an independent risk factor for the development of atrial fibrillation, LA thrombus and subsequent stroke. The aim of this study was to measure LA electromechanical dissociation (EMD), LA volumes, P-wave dispersion (PWD) and P-wave terminal force in V1 (PTFV1) as markers of atrial cardiopathy in patients with ESUS (embolic stroke of undetermined source), to determine whether atrial cardiopathy is an integral part in the causal pathway of ESUS. 28 patients presenting with ischemic stroke and fulfilling the criteria for ESUS were enrolled into this cross-sectional, observational study along with a control group of 28 age- and gender-matched apparently healthy individuals. On ECG, PWD and PTFV1 were measured. On echocardiography, LA EMD and LA volumes were recorded. Increased PWD (34.14 ± 9.89 ms vs. 27.32 ± 8.95 ms; p = 0.01), atrial EMD (73.32 ± 16.31 ms vs. 63.63 ± 13.59 ms; p = 0.02) and LA volumes were observed in patients with ESUS as compared to controls. A significant correlation was also found between these parameters (p < 0.01). According to the results of our study, PWD, atrial EMD and LA volumes may be novel predictors for ESUS. Our results support the notion that atrial cardiopathy is a distinct mechanism of thrombosis in ESUS patients. Further research is required to clarify its function in the causation of stroke, ESUS in particular.

## Introduction

Roughly one-third of all cases of ischemic stroke divulge no discernible cause despite standard evaluation and are referred to as cryptogenic strokes. Extensive research on such patients led to the introduction of the acronym ESUS, which stands for ‘Embolic Stroke of Undetermined Source’, by Robert Hart and colleagues of the Cryptogenic Stroke/ESUS International Working Group, to include cases of non-lacunar ischaemic stroke with a probable embolic source, allowing anticoagulation in this subset to be a distinct treatment option^1^. Data shows that such ESUS patients are said to account for approximately 19% of all patients of ischaemic stroke^2^ and are generally younger than patients suffering from ischemic stroke due to other causes^2–4^. These patients also have higher recurrence rates of stroke, as compared to the other ischemic stroke counterparts, further compounding the need for a definitive treatment regimen for secondary prevention in such patients^5–8^.

ESUS patients comprise a heterogenous population with regards to aetiology. In a recent study, 3 stroke registries were examined to include 800 patients with ESUS, revealing that the most common potential sources for embolism in these patients include left ventricular disease (54.4%), arterial disease (48.5%) and atrial cardiopathy (45%). It was also observed that in a majority of these patients there existed more than one potential embolic source^9^.

Recent data eroding the evidence for the temporal association between atrial fibrillation and ischaemic stroke have indicated that cardiac structural changes independent of AF may be on the causal pathway for the development of thromboembolism. The results further the case for viewing the mechanism of embolism in ESUS patients as more complex and multi-faceted, suggesting that atrial fibrillation and atrial cardiopathy may not be part of a continuum, rather exist on a spectrum of events, with the implication that the presence of one may not hinge on the absolute presence of the other.

Recently, atrial cardiopathy, a condition characterized by structural, functional, and biochemical abnormalities of atria prone to fibrillation, has emerged as a possible pathogenic mechanism in ESUS. Many electrocardiographic (ECG) and echocardiographic markers have been proposed in order to detect an altered atrial substrate at an early stage^10^. ‘Atrial failure’ is a clinical term that is also being brought into common parlance, and refers to “any atrial dysfunction (anatomical, mechanical, electrical, and/or rheological, including blood homeostasis) causing impaired heart performance and symptoms, and worsening quality of life or life expectancy, in the absence of significant valvular or ventricular abnormalities”^11^. Further research may well show a significant contribution of this condition in the pathophysiology of cardioembolic stroke.

Our study was undertaken with the aim of observing the relationship between ESUS patients and the markers of atrial cardiopathy, in turn to establish whether atrial cardiopathy may be a direct cause for the development of stroke by virtue of the changes brought about in the left atrium, and whether the presence of AF may be a consequence of these changes rather than a cause, along with stroke. Furthermore, our study would help to establish these markers as predictors for stroke and may also contribute to the development of a management algorithm with regards to secondary prevention in appropriate patients of ischaemic stroke.

## Methods

### Study design

Our study was a cross-sectional, observational study undertaken in Jawaharlal Nehru Medical College and Hospital, Aligarh Muslim University, for a period of 20 months, enrolling 28 cases of ESUS and 28 age- and sex-matched controls. The study protocol was approved by the Board of Studies and cleared by the ‘Institutional Ethics Committee’, Aligarh Muslim University, Aligarh, Uttar Pradesh, in March 2018. Informed consent was obtained from all patients or their respective legal guardians. All research was performed in accordance with the Declaration of Helsinki.

### Study population

ESUS was defined as an infarct visualized by CT (computed tomography) or MRI (magnetic resonance imaging) brain that is not lacunar*, in the absence of (a) extracranial or intracranial atherosclerosis causing ≥50% luminal stenosis in arteries supplying the area of ischemia, (b) major-risk cardioembolic source, and (c) any other identified specific cause of stroke. The assessment required for the ESUS diagnosis included brain CT or MRI, 12-lead ECG, cardiac monitoring for ≥24 hours with automated rhythm detection, precordial echocardiography, and imaging of the extra- and intra-cranial arteries (catheter, MR, or CT angiography, or cervical duplex plus transcranial doppler ultrasonography).

*Lacunar stroke was defined as a subcortical infarct ≤1.5 cm (≤2.0 cm on MRI diffusion images) in the largest dimension and in the distribution of the small, penetrating cerebral arteries^1^.

Patients with lacunar infarcts, significant carotid artery disease, persistent AF, rheumatic heart disease or other structural heart disease or those with prosthetic heart valves were excluded from the study. In addition, all patients underwent 24-hour Holter monitoring to rule out the presence of AF.

### Investigations

#### ECG

- The onset of the P-wave was defined as the junction between the isoelectric line and the beginning of P-wave deflection. The offset was defined as the junction between the end of the P-wave deflection and the isoelectric line. The longest atrial conduction time measured on any of the 12 leads was defined as P maximum (Pmax) and the shortest time was defined as P minimum (Pmin). The difference between Pmax and Pmin was calculated and defined as P-wave dispersion (PWD = Pmax − Pmin)^12^.
- P-wave terminal force in lead V1 (PTFV1) was defined as the duration of the negative terminal deflection of the P-wave in lead V1 multiplied by the absolute value of its amplitude. Measurements were manually made from the admission standard 12-lead ECG recorded with the subject at rest in supine position at paper speed of 50 mm/s and calibration of 10 mm/mV. Increased PTFV1 was considered a value greater than 0.04 mm·s^13^.

#### Echocardiography

**Figure 1:**
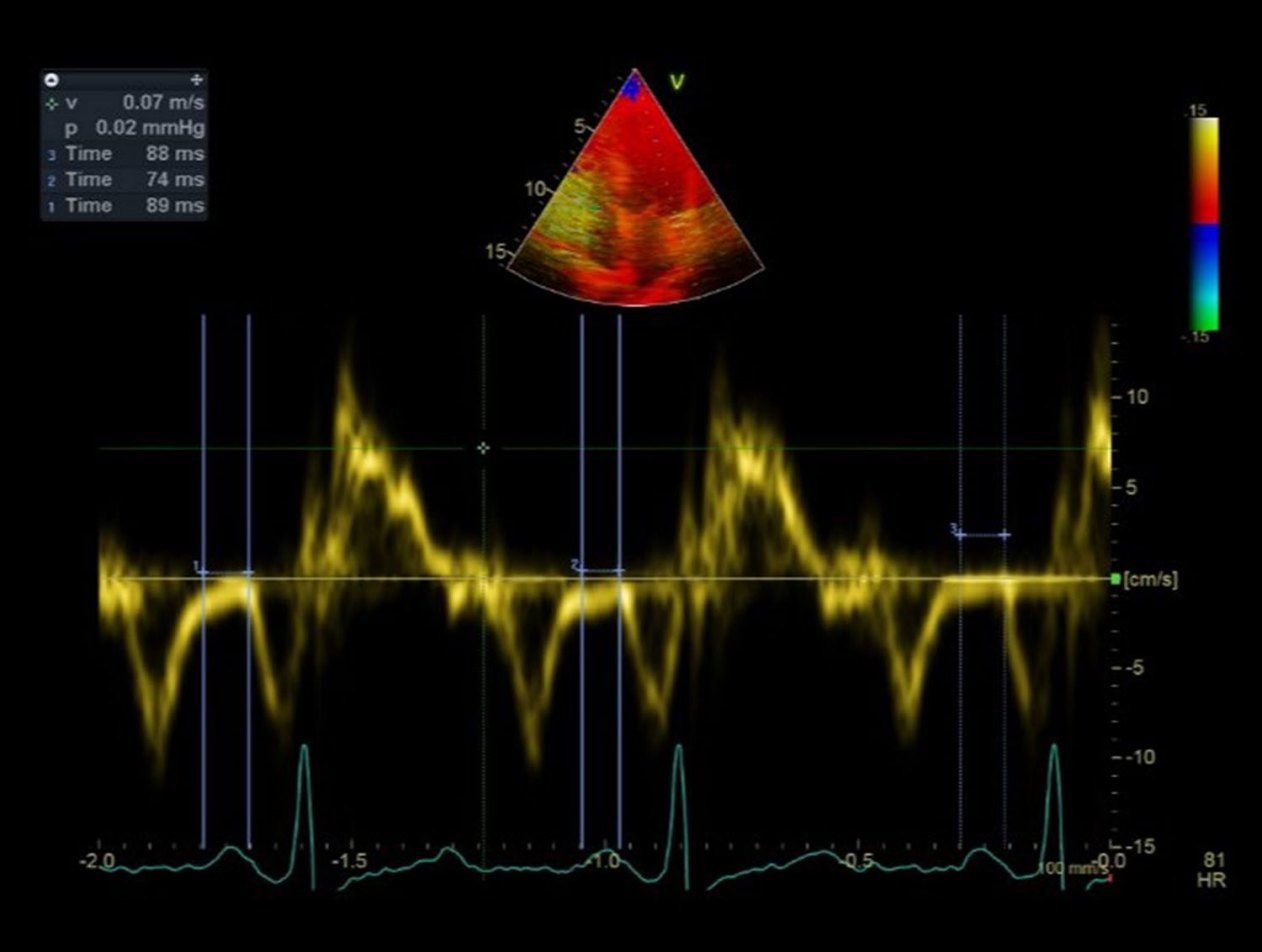
Left atrial electromechanical delay measurement in a study participant.

- Time intervals from the beginning of P-wave to beginning of A´ wave from the lateral mitral annulus in tissue doppler imaging was recorded as the intra-atrial electromechanical delay (Fig. 1). Average values of these indexes obtained from 3 consecutive cardiac cycles were used for analysis^14,15^.
- LA volumes using the Modified Biplane Simpson’s method in the apical four- and two-chamber views were also measured, as mentioned:
  - LA passive volumes consisting of:
    - Pre-atrial contraction volume (LAV_preA_): measured at the onset of the P-wave on an electrocardiogram (ECG);
    - Minimal LA volume (LAV_min_): measured at the closure of the mitral valve in end-diastole; and
    - Maximal LA volume (LAV_max_): measured just before the opening of the mitral valve in end-systole.
  - LA active volumes measured include:
    - LA reservoir volume (LAV_max_ − LAV_min_)
    - LA passive emptying volume (LAV_max_ − LAV_preA_)
    - LA contractile volume (LAV_preA_ − LAV_min_)

All volumes were indexed to body surface area (BSA) and expressed in mL/m^2^.

### Statistical analysis

All statistical data was analysed by using SPSS Software Version 20 for Windows. Continuous variables were expressed as mean ± standard deviation while proportions were expressed as count (percentages). Comparison of categorical variables between the groups was done by the Chi-square test while continuous variables were compared using the student ‘t’ test for independent groups. Non-parametric tests were used wherever appropriate. Pearson’s test was used for correlation analysis. Receiver operating characteristic (ROC) curve analysis was used to determine the optimum cut-off level of P-wave dispersion, EMD and LA volumes to predict ischaemic stroke. A two-tailed ‘p’ value of less than 0.05 was considered significant.

### Conference presentation

The abstract of a previous version of this paper was selected for display in the 2018Stroke ePosters’ category with the title ‘Evaluation of left atrial mechanics and p-wave dispersion as markers of left atrial cardiopathy in patients with embolic stroke of undetermined source (ESUS)’ in the ESC CONGRESS 2020 – The Digital Experience^16^.

## Results

Among the 28 patients with ESUS recruited to the study, the mean age was observed to be 51.57 ± 15.92 years, out of which 61% (n = 17) were males, with maximum number of patients falling into the age group of 41–60 years (53%). The baseline demographic and laboratory characteristics were similar between the case and control groups. There were no significant differences regarding age, gender, and body surface area between patient and control groups (p > 0.05). Variables like hypertension, diabetes and smoking were also equally distributed between the study groups (Table 1).

**Table 1:** Baseline characteristics of study population.

Finding	Case	Control	p value
Age (years)	51.57 ± 15.92	48.50 ± 13.61	0.44
Male (n)	17 (61%)	16 (57%)	0.78
Hypertension (n)	17 (61%)	13 (46%)	0.28
Diabetes (n)	7 (25%)	5 (18%)	0.51
Smoking (n)	12 (43%)	9 (32%)	0.4
Previous stroke (n)	9 (32%)	–	–
Body surface area (m^2^)	1.76 ± 0.07	1.75 ± 0.06	0.87

With regards to the variables of interest in our study (Tables 2 and 3), we observed that the mean P-wave dispersion in the case group was higher than that in the control group (34.14 ± 9.89 ms vs. 27.32 ± 8.95 ms; **p = 0.01**). Using a cut-off level of 36 ms, P-wave dispersion predicted stroke with a sensitivity of 54% and specificity of 86% (ROC area under the curve: 0.700, 95% CI 0.561–0.838, p = 0.01). However, there was no significant difference in the value of P-wave terminal force in V1 in the case and control groups (0.0156 ± 0.022 vs. 0.0184 ± 0.019; p = 0.61).

**Table 2:** LA parameters in the case and control groups.

Finding	Case	Control	p value
P-wave dispersion (ms)	34.14 ± 9.89	27.32 ± 8.95	**0.01**
Electromechanical delay (ms)	73.32 ± 16.31	63.63 ± 13.59	**0.02**
Pre-atrial contraction volume (ml)	23.94 ± 7.66	17.93 ± 2.18	** < 0.01**
Minimal LA volume (ml/m^2^)	17.01 ± 8.55	10.09 ± 0.97	** < 0.01**
Maximal LA volume (ml/m^2^)	35.51 ± 8.21	29.76 ± 1.92	** < 0.01**
LA reservoir volume (ml/m^2^)	18.49 ± 2.09	19.66 ± 1.34	**0.02**
LA passive emptying volume (ml/m^2^)	11.56 ± 1.59	11.82 ± 1.78	0.56
LA contractile volume (ml/m^2^)	6.92 ± 1.59	7.83 ± 1.9	0.06
PTFV1 (mm·s)	0.0156 ± 0.022	0.0184 ± 0.019	0.61

Significant values are in bold.

**Table 3:** Receiver operating characteristics (ROC) curve of study parameters for predicting atrial cardiopathy in ESUS patients.

ROC of the study parameters				
Parameter	Cut-off value	Sensitivity	Specificity	Youden index
P-wave dispersion (ms)	36	0.536	0.857	1.393
EMD (ms)	79.00	0.464	0.929	1.393
LAV_preA_ (ml/m^2^)	18.7223	0.75	0.786	1.536
LAV_min_ (ml/m^2^)	11.3155	0.75	0.929	1.679
LAV_max_ (ml/m^2^)	30.2463	0.679	0.679	1.358
LA reservoir volume (ml/m^2^)	20.5648	0.179	0.857	1.036

The mean left atrial EMD in the case group was also found to be higher than the control group (73.32 ± 16.31 ms vs. 63.63 ± 13.59 ms; p = 0.02). At a cut-off level of 79 ms, atrial EMD predicted stroke with a sensitivity of 46% and specificity of 93% *(ROC area under the curve: 0.680, 95% CI: 0.539–0.822, p* = *0.02)*.

Among the LA volumes, the pre-atrial contraction volume (23.94 ± 7.66 vs. 17.93 ± 2.18 ml/m^2^; p < 0.01), LA minimal volume (17.01 ± 8.55 vs. 10.09 ± 0.97 ml/m^2^; p < 0.01), LA maximal volume (35.51 ± 8.21 vs. 29.76 ± 1.92 ml/m^2^; p < 0.01) were significantly increased in the case group as compared to the control group, while the LA reservoir volume (18.49 ± 2.09 vs. 19.66 ± 1.34 ml/m^2^; p = 0.02) was found to be markedly decreased in the case group. The cut-off levels of LA volumes with regards to prediction of stroke are mentioned in Table 3.

A significant correlation was found for P-wave dispersion with EMD (r = 0.425; 95% CI: 0.147–0.644, p < 0.01), LA maximal volume (r = 0.408; 95% CI: 0.226–0.551, p < 0.01), LA minimum volume (r = 0.411; 95% CI: 0.262–0.561, p < 0.01) as well as pre-atrial contraction volume (r = 0.399; 95% CI: 0.219–0.547, p < 0.01) in our study for the prediction of stroke. The mean EMD was also found to positively correlate with the mean values of LA maximal volume (r = 0.354; 95% CI: 0.068–0.565, p < 0.01), LA minimum volume (r = 0.386; 95% CI: 0.116–0.566, p < 0.01) and pre-atrial contraction volume (r = 0.356; 95% CI: 0.069–0.553, p < 0.01). The overall regression model for EMD, P-wave dispersion, LAV_preA_, LAV_min_ and LAV_max_ was significant [F(5, 50) = 4.55, p < 0.01, R^2^ = 0.31].

## Discussion

**Figure 2:**
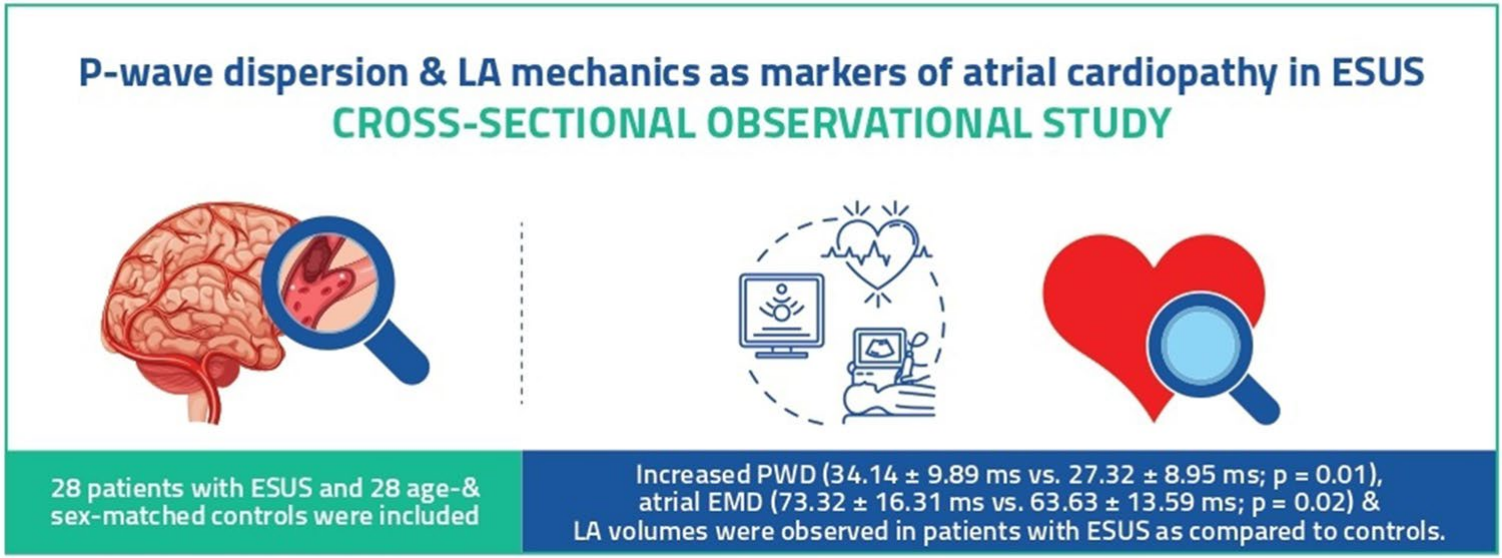
Graphical abstract.

We observed increased values of P-wave dispersion, LA electromechanical delay and LA volumes in patients with ESUS in our study *(*Fig. 2*)*. Various studies have shown that inter- and intra-left atrial EMD are likely independent predictors for the development of AF and stroke^15,17^. P-wave dispersion is a variable that has been of much interest with regards to its presence in patients suffering from cardioembolic stroke, and multiple studies have found a definite association between them^18,19^. Likewise, one study comparing PWD and atrial electromechanical delay between healthy elderly (75.4 ± 6.9 years) and a younger control group (42.7 ± 9.6 years) found increased values of both in the elderly group, without any evidence of AF in either group, which signifies that a natural progression to AF in the elderly may involve a similar pathway as that hypothesized in ESUS patients. Aging, which in itself is a risk factor for AF, was found to be correlated with increased left atrial size and impaired diastolic relaxation, which is not dissimilar to the changes also seen in patients with atrial cardiopathy. In fact, a study revealed that patients who underwent intensive vascular risk factor management after catheter ablation of AF had a significant reduction in left atrial size and a lower rate of AF recurrence than patients whose risk factors were not managed as intensively^20^. This indicates that the management of AF alone may not be the crux point for prevention of stroke, rather it could be more beneficial to interrupt the processes involved in the pathophysiology of thrombus generation, which include risk factors like ageing, obesity, alcohol, amongst others. This may also explain the association that has been found in multiple studies between markers of atrial cardiopathy and various conditions like obesity, psoriasis vulgaris, PCOS etc.^21–26^.

Similar to our study, investigators have found that left atrial enlargement is an independent risk factor for ischemic stroke, especially of the recurrent form, which is more commonly seen in patients with ESUS^27^. Recent similar studies have also established this association in patients with ESUS, suggesting echocardiogrpahic parameters to be of interest in ESUS population^28^. Increased mean LA volume indices (LAVI) are associated with the cardioembolic phenotype of ischemic stroke^29^, while one study found that increased LAVI significantly related to stroke recurrence in patients with non-sustained atrial tachycardias but without previously documented AF^30^. These findings impart weight to the discovery of the association between stroke and other atrial dysrhythmias besides AF^31–33^. In a study including 111 patients with ischaemic stroke, increased LA fibrosis and lower LA ejection fraction, as observed on cardiac MRI, were detected with similar incidence in patients with undetermined cause of embolism and those with underlying AF, further denoting that an underlying atrial disease could very well be the origin of the embolic event^34^.

The rationale for the temporal association between stroke and AF has been paradoxically diminished in view of recent studies demonstrating a surprising absence of AF in a majority of patients monitored before the index event of cardioembolic stroke^35,36^. Furthermore, the transient nature of AF led to prolonged rhythm monitoring in patients with stroke of unknown aetiology, however long-term follow-up found that only 30% of cryptogenic stroke patients manifested any AF even after 3 years of continuous heart rhythm monitoring via an implantable loop recorder^37^.

Our study was unable to find a significant difference in the value of PTFV1 between case and control groups. P-wave terminal force in V1 has been found to be associated with cryptogenic stroke and underlying AF in multiple studies^13,38^, however it is interesting to note that a study by Sajeev et al. consisting of 435 patients with ischaemic stroke in the absence of AF and other causes, found that morphology consistent with PTFV1 on ECG occurred commonly in both the stroke/TIA and control groups. There was no significant difference in the median PTFV1 value between the stroke 3.96 mV·ms [Interquartile range (IQR) 2.78–5.58] and control 4.23 mV·ms [IQR 2.91–5.57] groups^39^. The authors noted that the measurements of PTFV1 demonstrated excellent intra-observer reliability on assessment of the same P-wave (Intra class correlation (ICC) 0.91, p b 0.001) with narrow limits of agreement 2.21 to − 2.95 mV·ms, however, a change in the P-wave assessed led to a significant reduction in reliability (ICC 0.79, p b 0.001). It is difficult to pinpoint whether the lack of correlation between ESUS and PTFV1 in contrast to the strong association found between AF and PTFV1 is due to the fact that PTFV1 represents specifically an atrial dysrhythmia state rather than an underlying diseased state; or rather due to inherent reliability issues with the marker, keeping in view the absence of a normal reference range as well as a dearth of sufficiently comprehensive methodology to allow for reproducible measurements of PTFV1, particularly in the presence of subtle baseline and beat to beat P-wave variability.

Our study was a single-centre study with a small sample size and thus additional extrapolation of our findings are limited until further confirmation by large-scale multi-centric studies. The lack of established methodology and reference ranges for the tested parameters can only be rectified by comprehensive investigations. Further research is required to cement these findings so as to develop normal ranges, as well as guidelines regarding testing these parameters in the appropriate subset of patients with cardioembolic stroke.

An overview of the existing evidence suggests that a case can be made for atrial cardiopathy being causally linked to the development of stroke due to the underlying cardiac structural changes, irrespective of the presence of dysrhythmia. A study found increased prevalence of atrial cardiopathy in patients with ESUS, with 26.6% of ESUS patients suffering from atrial cardiopathy (defined as severe LAE on echocardiogram or PTFV1 > 5,000 μV-ms on ECG), compared to only 12.1% of stroke patients with large artery atherosclerosis (LAA) and 16.9% of those with small vessel disease (SVD) (p = 0.001)^40^. Another similar study demonstrated increased incidence of atrial cardiopathy (defined by severe left atrial enlargement (sLAE) in patients with ESUS as compared to patients with non-cardioembolic strokes^41^. A recent study postulates that transient atrial mechanical dysfunction could be a part of the pathophysiology in patients with ESUS^42^.

The concept of atrial cardiopathy may clarify why the onset of AF occurs at or around the time of incident stroke in many cases, conveying that thromboembolism and dysrhythmia both develop in parallel as part of the progression of an underlying atrial cardiopathy^43^. These considerations would also help to explain the results of a study in which only 31% of patients with both subclinical atrial fibrillation and stroke had no AF during a median 8 months of continuous heart-rhythm monitoring before the stroke and only manifested AF after the stroke^35^.

These suppositions beget the question of whether the presence of reliable indicators of atrial cardiopathy may be feasible in the diagnosis and treatment algorithm of embolic stroke and, furthermore, if they may even help to predict the development of events like AF and cardioembolic stroke (CES). In fact, a study has already demonstrated that in acute ischemic stroke patients without a pre-existing diagnosis of AF, the odds of suffering from CES is associated with changes in structural and functional measurements demonstrated on routine stroke care TTE. The most significant association was seen with increases in LA systolic diameter^44^.

Our findings demonstrate that changes in echocardiographic parameters may be reflective of a dynamic process in the structure of the atria with the consequent development of atrial cardiopathy and are independently associated with a diagnosis of ESUS and are not simply markers of pre-existing AF. Moreover, the correlation that we observed between the various study parameters also hint that these aberrations signify an overall process of atrial disease that may be manifest more readily in a specific subset of patients in whom anticoagulation may very well be an appropriate component in the management of stroke. The parameters in our study have the added advantage of being non-invasive and relatively cost-efficient, and thus could prove to be more valuable in the diagnostic workflow if employed in the relevant scenarios.

## Data Availability

The datasets generated and/or analysed during the study are available from the corresponding author on reasonable request.
